# Draft genome of co-cultured *Melainabacteria* sp. 17Bon1m

**DOI:** 10.1128/mra.01002-23

**Published:** 2024-01-11

**Authors:** Heidi Abresch, Sophia Miller, Kathryn Bick, Scott Miller

**Affiliations:** 1 Division of Biological Sciences, University of Montana, Missoula, Montana, USA; California State University San Marcos, San Marcos, California, USA

**Keywords:** *Melainabacteria*

## Abstract

We report the 7.6 Mb draft genome sequence of *Melainabacteria* sp. strain 17Bon1, which was sequenced from a co-culture with the diatom *Rhopalodia gibba* collected from the Clark Fork River in Bonita, MT.

## ANNOUNCEMENT

*Melainabacteria* are the closest non-photosynthetic relatives of cyanobacteria but are poorly understood due to difficulties with laboratory cultivation (1, 2). Although they have been identified in metagenomes across the globe, *Melainabacteria* are currently understood from metagenome-assembled genomes and not from laboratory-grown cultures. Recently, DNA sequencing revealed that a member of *Melainabacteria* (*Melainabacteria* sp. 17Bon1m) was growing in culture together with the diatom *Rhopalodia gibba,* which came from a sample collected from the Clark Fork River in Bonita, MT, in August 2017. *R. gibba* was isolated through repeated rounds of single diatom cell transfer from 1.0% CSi-N agar plates to 20-mL tubes containing CSi-N media. The culture is maintained in CSi-N liquid media at 20°C with a 12:12 hour light:dark cycle.

DNA was extracted from separate culture flasks in September and October 2018 using the Qiagen DNeasy PowerBiofilm kit according to manufacturer instructions. Genomic libraries were prepared using the Nextera Flex DNA Library kit, and paired-end sequencing was performed with Illumina MiSeq (Reagent Kit v2, 500 cycles) over three runs, resulting in 40,405,158 reads in total. High-molecular-weight DNA was extracted in April 2019 using the protocol described in Miller et al. (3), prepared with a MinION Ligation Sequencing Kit without size selection, and sequenced by Nanopore using a FLO-MIN106 flow cell resulting in 1,032,503 reads ranging from 132 to 24,785 bp in length. Read quality was assessed using FastQC v0.11 (https://www.bioinformatics.babraham.ac.uk/projects/fastqc/).

A hybrid metagenomic assembly was created with SPAdes v3.12.0 (4) using the --meta option. Scaffolds <1,000 bp long or <1× coverage were removed from the assembly before further curation. Putative *Melainabacteria* sp. 17Bon1m scaffolds with high sequence similarity to previously sequenced *Melainabacteria* sp. BJP_IG3402_49_16 (NCBI accession: GCA_003242885) were identified using BLAST+ v2.2.31 (5) using default parameters. These putative scaffolds were further manually refined using online BLAST databases on the NCBI website. Scaffolds were retained if they met the following conditions: coverage of ~35× (or a multiple thereof), a top BLASTn hit to a *Melainabacteria* sequence with greater than 70% sequence identity, and an *E*-value greater than 10^−10^. Scaffolds not within these values or with no significant similarities found using BLASTn were checked using BLASTx, whereby scaffolds that had the highest similarity to *Melainabacteria* sequences were retained.

**Fig 1:**
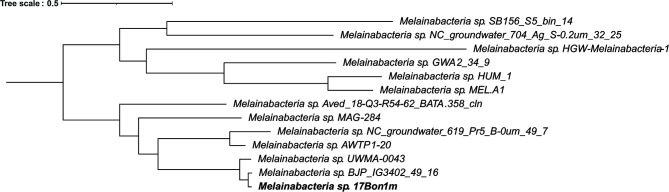
A maximum likelihood phylogeny reconstructed from a concatenated alignment of 80 single-copy orthologous amino acid sequences in *Melainabacteria* metagenomes. Constructed in IQTree using the LG+F+I+G4 model and 1,000 bootstrap replicates. Bootstrap values at all nodes = 100. The tree was visualized using the Interactive Tree of Life tool (11). The *Melainabacteria* sequence presented here is highlighted in bold.

This filtering resulted in 35 scaffolds identified as *Melainabacteria* sp. 17Bon1m, with a total length of 7,554,073, scaffold N50 value of 768,404 bp, GC content of 49.5%, and mean coverage of 35.0×. Genomic statistics for these scaffolds were assessed using QUAST v4.5 (6). We used OrthoFinder (7) with the parameters -M msa -oa to identify and align 80 shared single-copy orthologs across 13 species of *Melainabacteria,* which were then concatenated by the program. Using IQtree v1.7-beta9 (8) with parameters -bb 1000 -alrt 1000 -m TEST, we constructed a maximum likelihood phylogeny with the LG+F+I+G4 model of amino acid evolution selected by ModelFinder (9, 10). *Melainabacteria* sp. 17Bon1m is most closely related to *Melainabacteria* sp. MAG BJP_IG3402_49_16 sampled from deep groundwater in Japan. (Fig. 1) The average nucleotide identity between 17Bon1m and the Japanese MAG is 90.88%.

## Data Availability

These data are associated with BioProject accession number PRJNA690824 under BioSample accession number SAMN36341331. The *Melainabacteria* sp. 17Bon1m assembly is available through GenBank accession number GCA_032128875.1. Raw data from Illumina sequencing are available under SRA accession numbers SRR26035200 and SRR26035201. Nanopore reads are available under SRA accession number SRR26035202.
